# Effect of vaginal misoprostol on pregnancy rate after intrauterine insemination: a randomized controlled trial

**Published:** 2015-01

**Authors:** Ziba Zahiri sorouri, Maryan Asgharnia, Ameneh Gholampoor

**Affiliations:** *Reproductive Health Research Center, Department of Obstetrics and Gynecology, Guilan University of Medical Sciences, Rasht, Iran.*

**Keywords:** *Misoprostol*, *Pregnancy*, *Intrauterine insemination*, *Randomized clinical trial*

## Abstract

**Background:**

Intrauterine insemination (IUI) is one of the most appropriate and cost-effective methods in infertility treatment.

**Objective:**

We aimed to investigate effect of vaginal misoprostol on pregnancy rate after IUI.

**Materials and Methods:**

Two hundred and ten infertile women who were referred to Infertility Clinic of Alzahra Hospital by an indication of IUI during 2012-2013 were randomly assigned to receive 200 µg vaginal misoprostol (n=105) or vaginal placebo (n=105) after IUI. For detecting pregnancy, past 2 weeks, beta human chorionic gonadotropin evaluation was made and if positive, transvaginal sonography was done for evaluation of pregnancy 2-3 weeks later and clinical pregnancy was recorded.

**Results:**

Pregnancy had been noted in 24 patients in misoprotol (22.9%) and 27 patients in placebo (25.7%) groups that this difference was not significant (p=0.748). In misoprostol group, 3 case of nausea and vomiting (2.9%) had been observed.

**Conclusion:**

According to the results, administering 200 µg vaginal misoprostol after IUI doesn’t have significant effect on the success rate of IUI.

## Introduction

up to now, based on the definite causes of infertility, various treatments such as ovulation induction, intrauterine insemination (IUI), in-vitro fertilization (IVF) and **intracytoplasmic sperm injection (ICSI)** have been applied and IUI had been indicated as one of the most suitable and cost effective methods which could be considered for idiopathic and male infertilities (1-5). In this method, semen should be processed and washed then sperm deposits directly in the uterine (3). 

Human semen contains large amounts of prostaglandins (PG) which are majorly produced by seminal vesicle. It had been demonstrated that there is less PG in males seminal fluid of infertile couples. Therefore, it seems that there is a relation between PG and pregnancy rate (6). In addition, it had been appeared that PGs could elevate rate of pregnancy by raising myometrial contractility, spermatozoon-oocyte binding and isthmic tubal relaxation, maintaining luteal phase and suppressing immune system (6). 

Also, prostaglandin E (PGE) could affect implantation (7). While standard IUI would consider only sperms, therefore, positive effects of other seminal agents on pregnancy would be eliminated (6). Misoprostol is an artificial analogue of natural PGE which could be applicable in many diverse issues (7). According to previous investigations there are controversial results regarding the effect of prostaglandins on the success rate of IUI (1, 6, 8). According to the results, rate of pregnancy by IUI varied widely (5-70% of IUIs could induce pregnancy) therefor diverse factors could affect its success rate (2, 5, 9-11). Distinguishing factors which could elevate the efficacy of IUI are helpful; therefore, we aimed to investigate effect of 200 µg vaginal misoprostol on pregnancy rate after IUI.

## Materials and methods

**Trial design and setting**

A parallel double blinded randomized placebo-controlled trial with equal randomization (1:1) was conducted on IUI candidate patients referred to the Infertility Clinic of Al-Zahra Hospital in Rasht, Iran during September 2012 to April 2013. 

**Patients**

Eligible patients were all women who were undergoing IUI and etiology of their infertility were male factor, idiopathic factors, and lack of pregnancy as a result of anovulation even after induction and producing dominant follicle, and other etiologies such as marital relationship problems. Medical and infertility histories had been collected. 

Causes of infertility were confirmed and classified by a gynecologist using physical examination and diagnostic procedures such as hysterosalpingography and laparoscopy for detecting tubal factor infertility. This study was approved by the Ethical Committee of Guilan University of Medical Sciences. All patients provided written informed consent before inclusion in the study.

**Randomization and interventions**

IUI cycle had been initiated during 1-3 days of menstrual period and vaginal sonography had been performed for all participants. In cases with the absence of >15 mm cyst, ovulation induction had been indicated and clomiphene citrate (IPSA-Switzerland) ±Human menopausal gonadotropin (HMG) (IPSA) was administered. Type and dose of medicines were administrated based on response to medicines monitored by serial transvaginal ultrasonography. 

Furthermore, serial transvaginal ultrasonography had been indicated to monitor follicles. By the presence of at least one dominant follicle ≥18 mm in size, 5000 IU HMG was injected and the absence of follicle leaded to exclude patients. Also detecting more than five follicles ≥13 mm in diameter or larger number (11 or more) of smaller follicles were cycle cancellation criteria. 

Thirty six hours following injection, patients again referred to Infertility Clinic to be prepared for IUI. Semen was prepared by the standard Swim Up technique in which liquefaction of seminal sample with buffer solution was performed in a sterile tube and centrifuged slowly at 200-300g for 10 min and the supernatant was discarded. This step was repeated 2-3 times and final remaining was over layered with 0.5-1 µg of culture medium and stored inside incubator at 37^o^C for 30-60 min to cause sperms with the most motility migrate to supernatant and be used for intrauterine injection. 

Before IUI, 0.1 mg of final swim up sample was placed on a pellet and sperm count and motility was calculated. Performing IUI was indicated in cases with at least 1 million sperms. Patients were randomly assigned in 2 groups using a random sequence that was prepared by a co-worker with no clinical involvement in the trial. The randomization list was generated using random number table. We provided two baskets labeled A and B contain sealed envelopes. After assignment, one envelope was delivered to gynecologist based on patient’s group. We put in each envelop one tablet. Tablet for misoprostol group was contained 200 µg of misoprostol and in the placebo group tablet with the same appearance had been applied. Misoprostol was produced by Samisaz Pharmaceutical Company, Iran, placebo was produced by Sobhan Darou Company, Iran.

Excess mucus which could block catheter was removed and the injection catheter with 0.5 ml sample was entered simply for 10-30 seconds in the cervical orifice and driven directly into uterine cavity. Immediately after IUI, vaginal misoprostol or placebo had been administered. Patients should be remained supine for 15 min after IUI. Patients were aware that intercourse, application of NSAIDs and antihistamines should be avoided from 72 hr before until 72 hr after insemination. In this trail, patients and gynecologist were blind of group allocation.

**Data collection and outcomes**

Primary outcome of this trial was clinical pregnancy rate after IUI. Investigators recommended immediate telephone contacts by detecting any complication. In addition, patients were asked to attend the Infertility Clinic 15 days after IUI and in the absence of menstrual period during past 2 weeks, beta human chorionic gonadotropin evaluation was made and if positive in these patients transvaginal sonography was done for evaluation of pregnancy 2-3 weeks later and clinical pregnancy was recorded. 

Fetal heart activities were defined as clinical pregnancy. Baseline data consisted age, body mass index (BMI), types and duration of infertility, motility and sperm count, and the number of dominant follicle during HMG injection were collected. Also adverse effects were assessed during 2 weeks after IUI. 

**Sample size**

According to previous investigations respectively with an error probability of 5% and a power of 80%, 105 participants in each group was necessary.

**Statistical analysis**

Data were analyzed in SPSS software (Statistical Package for the Social Sciences, version 21.0, SPSS Inc, Chicago, Illinois, USA). Fisher exact test and Chi- square to compare frequency of qualitative variables and independent T-test to compare means between two groups were used. P-value<0.05 was considered to indicate statistical significance.

## Results

**Figure 1:**
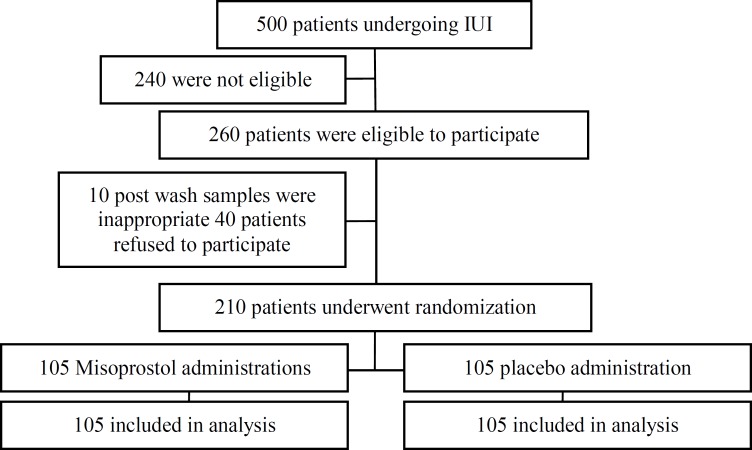
Flow of participants

During study period from 260 infertile women who were eligible, 40 patients refused to participate and 10 patients were excluded because post wash samples were inappropriate. Two hundred and ten patients were randomly assigned with equal number in two 200 µg misoprostol (n=105) and placebo (n=105) groups. All patients completed the trial (Figure 1). 

Baseline characteristics of the patients are shown in table I. There were no significant differences between groups. Pregnancy had been noted in 24 (22.9%) patients of misoprostol and 27 (25.7%) patients of placebo groups which revealed no significant difference between groups (p=0.748). Nausea and vomiting had been noted in 3 (2.9%) misoprostol patients and no other adverse effects such as hemorrhage, cramp or abdominal pain was noted in groups.

**Table I:** Baseline characteristics of patients

**Variables **			**200 µg ** **misoprostol**	**Placebo**	**p-value**
Mean age (year)			31.01 ± 5.43	29.59 ± 5.41	0.059 a
Mean BMI			28.11 ± 6.33	28.02 ± 7.56	0.930 a
Types of infertility					
	Primary		89 (84.76)	96 (91.42)	0.200 b
	Secondary		16 (15.24)	9 (8.57)	
Etiology of infertility					
	Male factor		18 (17.14)	8 (7.62)	0.055 b
	Ovarian factor		42 (40.00)	58 (55.24)	
	Male and ovarian factors		13 (12.38)	8 (7.62)	
	Idiopathic and other factors		32 (30.48)	31 (29.52)	
Mean duration of infertility (year)			4.06 ± 3.10	3.56 ± 3.04	0.240 a
Sperm motility					
	Normal		93 (88.57)	92 (87.62)	1.0 b
	**Astenospermia**		12 (11.43)	13 (12.38)	
Sperm count (×10^6^/ml)					
	Normal		90 (85.71)	98 (93.34)	0.113 b
	Oligospermia		12 (11.43)	7 (6.66)	0.336 b
	Severe oligospermia		3 (2.86)	0	0.246 b
Mean dominant follicles			5.22 ± 2.46	4.63 ± 2.70	0.099 a

N=105

* Data are Mean ± SD or n(%).

a : Independent t-test was used.

b : chi-square test was used.

## Discussion

PG exists in all body fluids and seminal fluids. The total amount of PG in seminal fluid is averagely 1mg which consisted of PGE, PGF and 19 hydroxylate PG. However, PGE with average amount of 2-27 µg/ml could be indicated as the chief prostaglandin (6). From 1947 which investigators assessed the role of decreased level of prostaglandin on idiopathic infertility for the first time, other investigators consequently concerned that the level of PGE in couples with idiopathic infertilities were lower than normal (1, 6).

Based on our finding pregnancy rate between the two groups was not significantly different which is consistent with results of the most of previous studies. In previous study in Iran by Moslemzadeh *et al*, 66 women were randomly assigned to two 200 µg vaginal misoprostol and placebo groups. Incidences of chemical pregnancies were similar in both groups (18.2%). 

Also clinical pregnancies were detected in 15.15% in misoprostol and 18.2% in placebo groups that this difference was not significant (1). Also in Billiet *et al* study among 146 IUI cycles in misoprostol group 19 pregnancies (13%) and in placebo group among 164 IUI cycles 21 pregnancies (12.8%) were detected that the difference was no significant (8).

In a study in Turkey in 34 cycles as the control group and 33 cycles as study group 200 mg misoprostol was administered vaginally at the time of insemination. Rates of pregnancy were 13% in control group and 11% in misoprostol group that this difference was not significant (12). In other study by Chikkagowdra* et al* 600 IUI cycles randomly assigned with equal number into 200 µg vaginal misoprostol group and control group. There was no significant difference between two groups in term of pregnancy rate which was detected in 8.4% of misoprostol group and 9.6% of control group (13).

But in contrast with our findings, Brown *et al* in a study used 400 µg misoprostol vaginally at the time of IUI in 253 IUI cycles and placebo in 241 IUI cycles, which showed that the pregnancy rate in misoprostol group was significantly greater than placebo group (17% vs. 9%) (6). Also in Barroso *et al* study in 29 IUI cycles 200 µg misoprostol was used vaginally after IUI and 30 IUI cycles were in control group. Pregnancy rate in misoprostol group was 31% that was significantly higher than control group (20%) (14). As, intrauterine semen injection could provoke some adverse effects, researchers applied washed sperms in IUI technique which could eliminate PG during the process (1, 6). 

Therefore, previous investigations recommended using misoprostol which is an artificial analogue of PGE1 to induce an influence similar to natural seminal fluid and increase pregnancy rate (6, 14). In addition, in our study similar to most previous studies investigators adminstered 200 µg misoprostol and results indicated no significant difference between groups. However, brown *et al* administered 400 mg misoprostol accompanied with 5 mg triglyceride and obtained higher pregnancy rate in misoprostol group in comparison with placebo group, while according to higher dose of the drug, more adverse effects had been noted (6). 

In addition, patients with any types of infertility had been participated and they mentioned higher significant difference between idiopathic infertility and uterine and pelvic infertilities which was inconsistent with our protocol which excluded uterine and pelvic factors. Also, in a review article by Chaninarong *et al* which investigated application of misoprostol during IUI, results showed that there was no significant result which advocates use of misoprostol to enhance pregnancy rate (7). Lack of assessing ovulation induction drugs (clomiphene± gonadotropin) could be addressed as limitations of this study.

## Conclusion

In conclusion, administration of 200 µg vaginal misoprostol after IUI has no significant effect on the success rate of IUI. Further investigations to provide evidences on low complication of misoprostol and promote performing investigations with higher doses of misoprostol could be recommended. 

## Note

***Registration ID in IRCT: IRCT201206191306N5***

***This article extracted from M.D. thesis. (Ameneh Gholampoor)***
